# Training effects of set- and repetition-interval rest time on recumbent-boxing exercise: Could virtual reality improve further?

**DOI:** 10.1016/j.isci.2023.107399

**Published:** 2023-07-20

**Authors:** Yi Wang, Qi Chen, Liangchao Liu, Qiuhong He, James Chung-Wai Cheung, Duo Wai-Chi Wong, Yang Liu, Wing-Kai Lam

**Affiliations:** 1 Department of Physical Education, Renmin University of China, Beijing 100872, China; 2 Sports and Social Development Research Center, Renmin University of China, Beijing 100872, China; 3 Physical Education Department, University of International Business and Economics, Beijing 100029, China; 4 School of Physical Education, Changzhou University, Changzhou 213164, China; 5 Department of Biomedical Engineering, Faculty of Engineering, The Hong Kong Polytechnic University, Hong Kong 999077, China; 6 Research Institute for Sports Science and Technology, The Hong Kong Polytechnic University, Hong Kong 999077, China; 7 L&L Technology PTY Company Limited, Cheltenham, VIC 3192, Australia; 8 School of Mechanics and Safety Engineering, Zhengzhou University, Zhengzhou 450001, China; 9 Sports Information and External Affairs Center, Hong Kong Sports Institute, Hong Kong 999077, China

**Keywords:** Health sciences, Computer science, Human-centered computing

## Abstract

This study examined the influence of set-interval and repetition-interval rest time of virtual reality (VR) boxing game in supine-lying posture. Fifty healthy middle-aged adults were randomly assigned into VR and non-VR groups to perform six different exercise protocols with varying set-interval and repetition-interval rest times (S_0_R_0_, S_0_R_1/3_, S_0_R_2/3_, S_40_R_0_, S_40_R_1/3_, and S_40_R_2/3_). Analysis on the non-VR group showed significant differences between exercise protocols for average heart rate (p < 0.001), maximum ventilation volume (p < 0.001), respiratory quotient (p < 0.001), oxygen pulse (p < 0.001), and excess post-exercise oxygen consumption (EPOC) (p = 0.003). VR appeared to have no further improvement on physical training effects in middle-aged adults, while the participants reported negative experience that might be associated with the over-exertion. Future study might need to explore game design elements that can accommodate high-exertion exercises.

## Highlights

• Recumbent-boxing exercises were conducted by middle-aged adults
• The different set-interval and repetition-interval rest times were evaluated
• Interval rest time significantly changed physical training effects
• Virtual reality could not improve the effects further

## Graphical abstract

**Figure:**
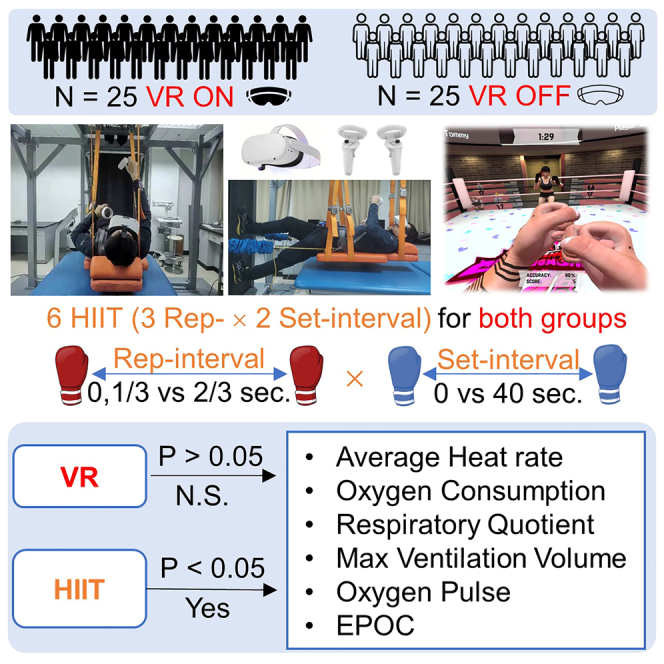

## Introduction

Physical inactivity is a global “pandemic” and is the fourth leading cause of death globally,^1^ particularly for older adults. Nearly half of the older adults, especially those living in residential care homes, achieved the recommended minimum level of physical activity for general health.^1^ The level of physical activity is strongly associated with the risk of chronic disease, mental illness, and cognitive and functional decline.^1^ Sufficient physical activity has an odds ratio of 0.83 for each additional chronic disease.^2^ In addition, higher levels of physical activity were associated with relative risks of 0.72 and 0.55, respectively, to dementia and Alzheimer’s disease.^3^ The World Health Organization (WHO) recommends older adults should perform regular muscle strengthening exercises and multi-component physical activity at moderate or greater intensity on three or more days a week, regardless of their level of mobility.^4^ Sustaining adequate physical activity levels could prevent nearly half of the deaths and the fact held that irrespective of whether an individual had a past habit of physical exercise.^5^

Mobility, functional limitations, and environmental constraints are key determinants to physical inactivity among older adults.^6^^,^^7^^,^^8^ Pain, discomfort, and functional dependence were physical barriers, while the concern of falling and the lack of self-confidence discouraged them from doing exercise, in addition to their psychosocial conditions.^9^^,^^10^ Environmental factors, including a lack of accessibility to equipment, care, and safe facility (such as handrails or caregiver support), affected the sense of security and thus contemplation of physical exercise among older adults.^6^ Small living place in some regions, such as Hong Kong, and restriction measures in care homes or nursing homes restricted their exposure to physical exercises,^11^ which worsened with the social distancing measures during COVID-19.^12^

One clinical application is required to promote the aerobic capacity and upper limb strength for the bed rest-bounded patients in hospital or elderly care home.^13^^,^^14^^,^^15^^,^^16^ Boxing training was selected as the main theme in this study as it is simple, is relatively safe (from acute injuries), is ready to use without gears, does not require professional training, and requires less space, which might be feasible for physical training during the pandemic.^17^ Boxing involves repetitive and continuous upper-body movements that impose high aerobic, metabolic, and cardiovascular demand on the body, despite that it is relatively static.^18^ Moreover, boxing-based high-intensity interval training (HIIT) is a time-efficient form of exercise and demonstrated high adherence rate and high anaerobic capacity compared to the HIIT in form of other sports.^19^ Synchronized movement that is enabled by HIIT might enhance the retention of training effects.^20^ Previous studies demonstrated that boxing training operated effectively with HIIT and might be better than an equivalent dose of brisk walking in improving body fat percentage, blood pressure, and oxygen consumption.^21^

The exercise prescription may consider several factors including exercise sequence, rest intervals between sets and exercises, frequency, velocity of movement, number of sets and repetitions, and load or intensity to match the specific training characteristics (e.g., maximal strength, localized muscular endurance, power). All of these factors can be manipulated to meet specific training goals and address individual needs.^22^^,^^23^ According to Fleck and Kraemer,^23^ the length of the rest interval between sets is an important variable when designing a resistance exercise program. The rest interval can play significant impact on acute and chronic metabolic, hormonal, and cardiovascular responses to resistance training.^22^^,^^23^^,^^24^ Previous studies that examined rest interval lengths from 1 to 5 min between sets for single exercises demonstrated significant differences in repetition performance and the exercise volume completed.^25^^,^^26^^,^^27^^,^^28^ However, these studies did not investigate the rest time between repetition interval on training. Furthermore, some patients are often unable to commit prolonged periods of training due to other concurrent training requirements.^29^ Therefore, an effective training strategy is required to attain an appropriate resistance training stimulus in a shorter period of time.^30^^,^^31^ All these warrant to compare the workout volume (repetition-interval and set-interval rest time) to determine the optimal training protocol.

Traditional health promotion interventions may not be sufficient to accommodate the situation, and it has been calling for creative thinking coupled with partnerships to promote physical activity in public.^32^ It is highly possible that virtual reality (VR)-based serious game (or exergame) could further improve the training effects. Previous reports suggested that the enjoyment in the game environment could offset fatigue and negative affective responses.^33^^,^^34^ The virtual game environment can also create positive experience to improve health and well-being, engaging older and inactive adults to do more exercise,^35^^,^^36^^,^^37^^,^^38^ and has been proven to be a valuable technique with success for the rehabilitation of cognitive behavior, motor coordination, pain relief, and life skills.^39^^,^^40^^,^^41^^,^^42^

To this end, we proposed a physical training program integrating boxing exercise with varying set-interval and repetition-interval rest time into VR technology in lie-down posture in hospital and care home. VR-driven exercise games (exergame) have been proven a valuable technique for the rehabilitation of cognitive behavior, motor coordination, and life skills.^39^^,^^40^^,^^41^ In addition to improving physical fitness, exercise games in the medium of VR can also create positive experience to improve health and well-being, engaging older adults to do more exercise.^35^^,^^36^^,^^37^^,^^38^ While traditional HIIT VR game was performed in a standing posture,^43^ our novelty of game design was to perform in a lie-down posture to accommodate the lack of room space or bed-bounded individuals with functional limitations. Similarly, in order to address the space and functional issues, VR exergames have also been created to be played while seated in an existing study.^44^

While different resting time between sets and repetitions may lead to distinct stimulation to aerobic and anaerobic systems, the objective of this study is to examine the influence of exercises protocols (in terms of set-interval and repetition-interval rest time) on the training effects of a recumbent-boxing exercise in middle-aged adults. As physical fitness in middle adulthood could promote active lifestyle that is associated with better health in their older ages,^45^^,^^46^ we considered healthy middle-aged participants in our pilot experiment instead of older adults to examine the efficacy and intensity level of VR exercises. In fact, middle-aged adults may start to experience physiological decline at the crossroads toward older age^47^ and may lack exercise habits due to various social challenges.^48^ HIIT VR exercises while lying in bed could promote an alternative exercise to help maintain muscle strength and cardiovascular responses, when the space and long exercise period is not available. It is expected that the research could promote their active healthy lifestyle toward their pre-older and older ages.

The secondary objective is to determine whether VR could further improve the training effects. We proposed to implement a lie-down (recumbent)-posture exercise with varying set-interval and repetition-interval rest time, which would impose different degrees of aerobic capability improvement. The findings from this study can provide insights of designing training exercise protocols in patients who were bed bounded.

## Results

### Basic information

The average height and body weight for all participants were 168.0 (SD: 3.3) cm and 65.8 (SD: 2.4) kg, respectively. The average height for VR and non-VR groups was 167.6 (SD: 3.5) cm and 168.4 (SD: 3.1) cm, while the average body weight was 65.5 (SD: 2.7) kg and 66.1 (SD: 2.0) kg, respectively. As shown in Table 1, we found no significant differences between the VR and non-VR group in age, body mass index, the maximum oxygen consumption level (VO_2_max), maximum ventilation volume (MVV), and oxygen pulse (p > 0.05).

**Table 1:** Comparison of basic information and baseline variables between VR and non-VR groups

Baseline Variables	Total, n = 50	VR, n = 25	Non-VR, n = 25	a*p* value
Age	45.10 (1.40)	45.28 (1.31)	44.92 (1.50)	0.370
BMI (kg m^−2^)	23.30 (0.51)	23.30 (0.49)	23.30 (0.55)	0.968
VO_2_max	2991.75 (145.94)	2967.33 (155.13)	3007.17 (137.55)	0.461
MVV	111588 (10232)	110497 (10888)	112680 (9629)	0.456
Oxygen Pulse	17.01 (0.96)	16.99 (1.05)	17.04 (0.89)	0.840

Results are presented in mean (standard deviation).

BMI: body mass index; MVV: maximum ventilation volume.

a p values calculated by independent sample t tests between the VR and non-VR groups.

**Figure 1:**
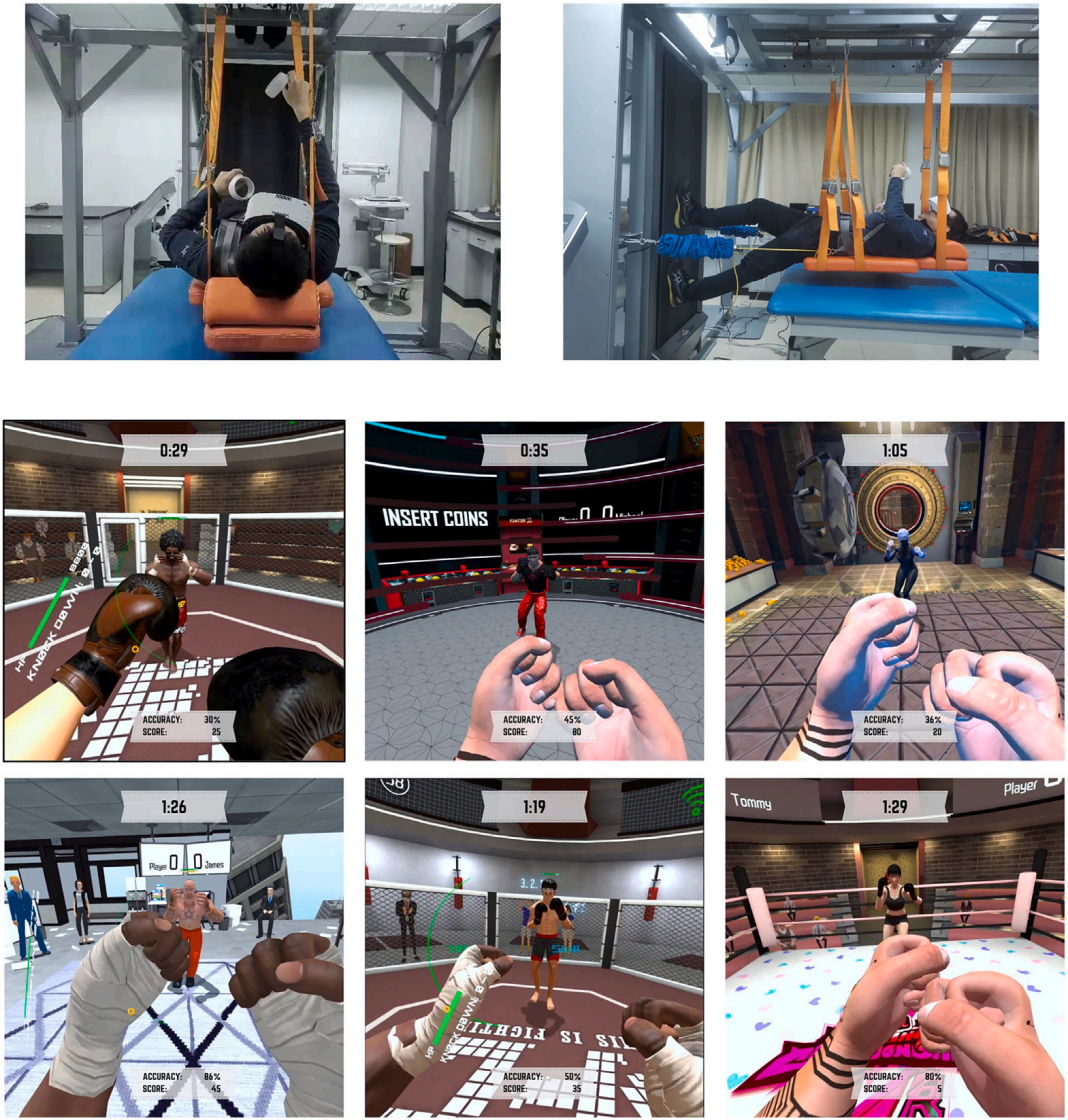
(Top) Configuration of recumbent-boxing posture; (Bottom) Virtual boxing environment and opponent avatar

The six exercise protocols involved the combination of two set-interval and three repetition-interval rest times. Set-interval rest time was set at either 0 s or 40 s. The repetition-interval rest time was set at 0 s, one-third seconds, or two-third seconds (denoted as: S_0_R_0_, S_0_R_1/3_, S_0_R_2/3_, S_40_R_0_, S_40_R_1/3_, and S_40_R_2/3_), as shown in Table 2. During the experiment, the participants were hanged on a supine recumbent position with the VR boxing game with self-selected fighting environments and opponent avatars (Figure 1).

**Table 2:** Arrangements of the six exercise protocols

Protocol	Set-interval rest time (s)	Repetition-interval rest time (s)	No. of sets	Punches per set	Set time (s)	Average punch speed (s^−1^)	Duration (min)
S_0_R_0_,	0	0	6	120	40	3	4
S_0_R_1/3_	0	1/3	6	120	80	1.5	8
S_0_R_2/3_	0	2/3	6	120	120	1	12
S_40_R_0_	40	0	6	120	40	3	8
S_40_R_1/3_	40	1/3	6	120	80	1.5	12
S_40_R_2/3_	40	2/3	6	120	120	1	16

### Influence of exercise protocols

**Figure 2:**
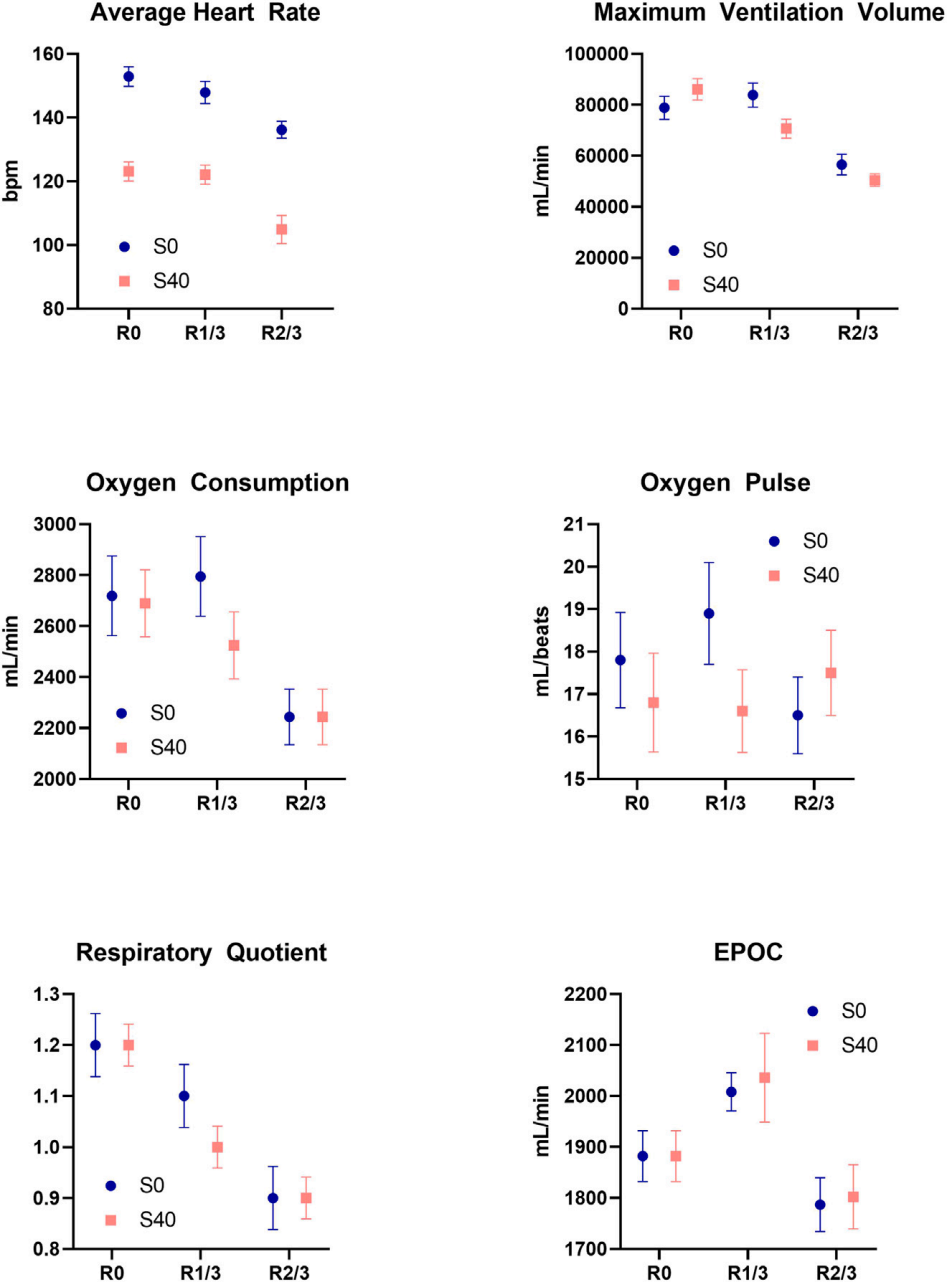
Cardiopulmonary measures of different exercise protocols in the non-VR group The error bars indicate standard deviation.

There were significant differences between exercise protocols for average heart rate (p < 0.001), MVV (p < 0.001), respiratory quotient (p < 0.001), oxygen pulse (p < 0.001), and excess post-exercise oxygen consumption (EPOC) (p = 0.003), while difference for oxygen consumption was not significant (p = 0.119). As shown in Figure 2, there were general trends that increasing rep-interval rest time lowered the average heart rate, MVV, oxygen consumption, and respiratory quotient. There was no apparent trend in effect of rep-interval rest time on oxygen pulse, while a repetition-interval rest time of 1/3 s seemed to induce higher EPOC. Moreover, a set-interval rest time of 0 s was consistently higher than that of 40 s for average heart rate.

**Figure 3:**
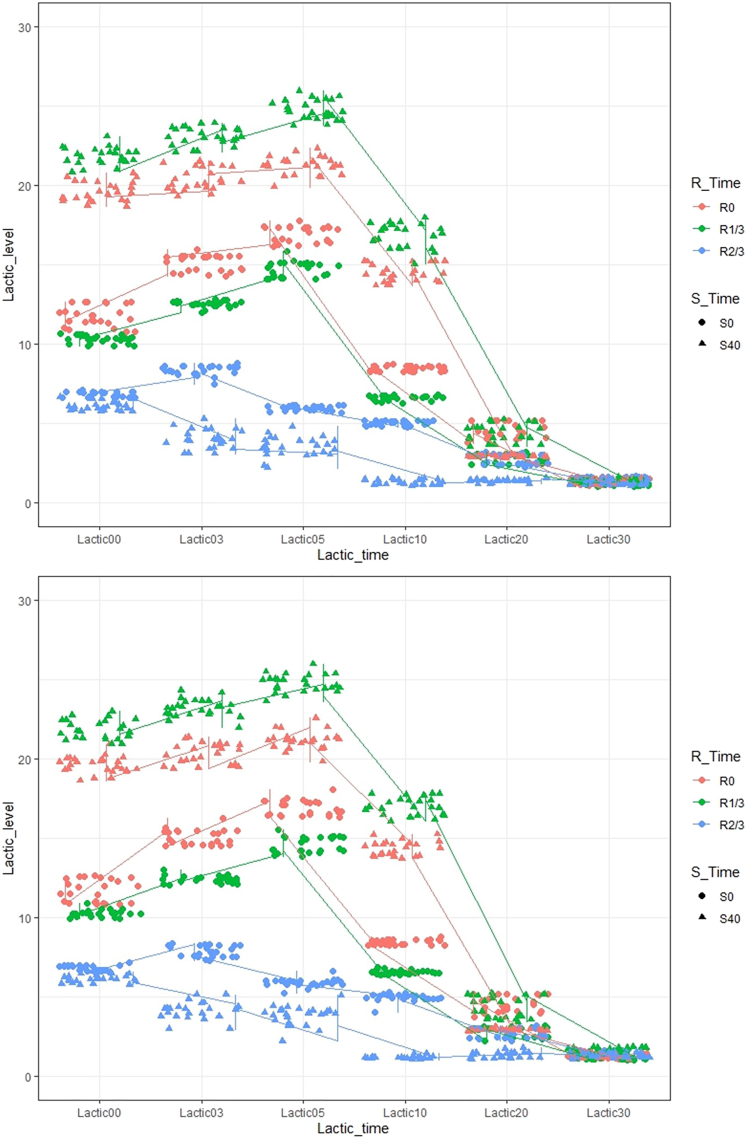
Overall trend of lactic acid level between VR (top) and non-VR (bottom) groups Unit: mmol/L.

Most of the pairwise comparisons demonstrated significant differences. An exercise protocol without set-interval (S_0_) and repetition-interval rest time (R_0_) induced the highest average heart rate at 153 (SD:3) beats per second. On the other hand, set-interval rest time at 40 s (S_40_) without repetition-interval rest time (R_0_) produced the highest MVV at 84,446 (SD: 4,703) L. Besides, there was a significant difference between exercise protocols on the overall lactic acid level (p value <0.001) (Figure 3).

### Influence of VR intervention

**Figure 4:**
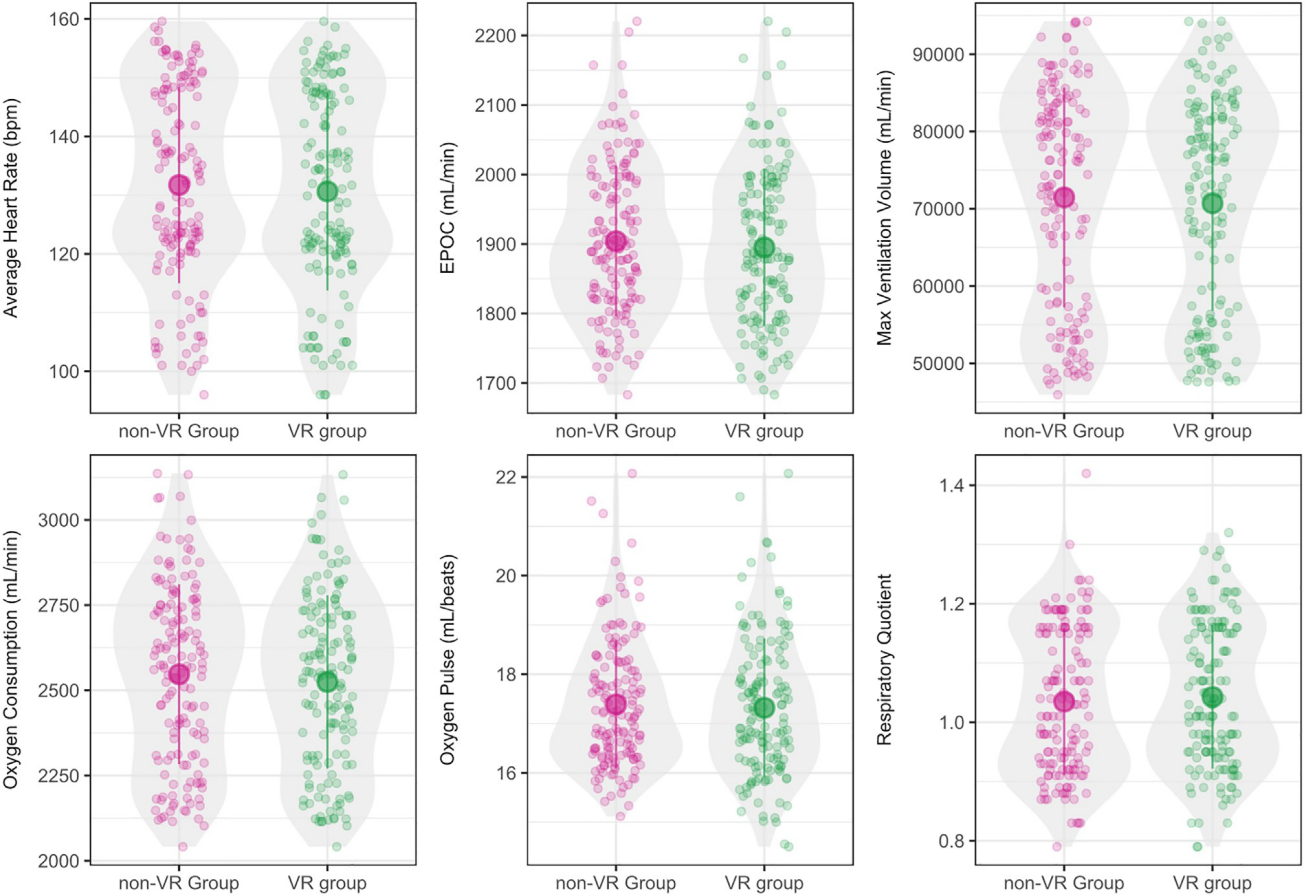
Differences of average heart rate, EPOC, maximum ventilation volume, oxygen consumption, oxygen pulse, and respiratory quotient between the VR and non-VR groups The error bars indicate standard deviation.

As shown in Table 3, VR intervention did not seem to induce any significant main effect or interaction effect for all cardiopulmonary measures (p > 0.05), while the significant effects of exercise protocol remained significant after incorporating the VR data in the generalizing estimating equation (p < 0.001). Figure 4 shows that cardiopulmonary measures between VR and non-VR groups were comparable.

**Table 3:** Model effects on cardiopulmonary measures, overall lactic acid level, and subjective feedback by questionnaires

Variable		Significance level (p value)		
		Interaction (Group × Protocol)	Group (VR vs. non-VR)	Exercise protocol
Cardiopulmonary Measures	Average heart rate	0.377	0.214	<0.001∗
	Oxygen Consumption	0.342	0.494	<0.001∗
	Respiratory Quotient	0.184	0.468	<0.001∗
	Maximum Ventilation Volume	0.289	0.447	<0.001∗
	Oxygen Pulse	0.150	0.783	<0.001∗
	EPOC	0.715	0.506	<0.001∗
Overall Lactic Acid		<0.001∗	0.155	<0.001∗
SVS	SVS	<0.001∗	<0.001∗	<0.001∗
IMI	Interest/enjoyment	0.009	<0.001∗	<0.001∗
	Perceived Competence	<0.001∗	<0.001∗	<0.001∗
	Pressure Tension	<0.001∗	<0.001∗	<0.001∗
	Effort Importance	<0.001∗	<0.001∗	<0.001∗
	Perceived Choice	<0.001∗	0.007	<0.001∗
	Value/usefulness	<0.001∗	<0.001∗	<0.001∗
	Relatedness	<0.001∗	0.587	0.574

For the subjective experience evaluated by the questionnaires, all of the constructs were significantly associated with VR, the exercise protocols, and their interaction, except relatedness from the Intrinsic Motivation Inventory (IMI). Compared to the non-VR group, the VR group demonstrated significantly lower Subjective Vitality Scales (SVS) and IMI scores in interest/enjoyment (median: 39 vs. 29), perceived competence (median: 34 vs. 23.5), and perceived choice (median: 28 vs. 26), but significantly higher pressure/tension, adjusted by the covariates, exercise protocols, and interaction in the generalized estimating equations (GEEs). In other words, the VR group performed worse in the aforementioned constructs. Nevertheless, VR reported a positive outcome in the value/usefulness (Table 4).

**Table 4:** Psychometrics measurement on subjective experience between the VR and non-VR group

Questionnaire Outcomes	VR Group	Non-VR Group
SVS	29 (24–32)	39 (36–41)
Interest/enjoyment	18.5 (15–22)	29 (24–32)
Perceived Competence	23.5 (19–28.25)	34 (31–36)
Pressure Tension	19 (16–22)	17 (14–19)
Effort/importance	34 (32–35)	30 (24–34.25)
Perceived Choice	26 (23–29)	28 (23–33)
Value/usefulness	31 (27–33)	28 (24–33)
Relatedness	23 (20–26)	23 (19.75–27)

Data presented as median (interquartile range); VR: Virtual Reality; SVS: Subjective Vitality Scale.

Besides, VR had no significant association with the overall lactic acid level (p = 0.155) despite that it significantly interacted with the effects of exercise protocol (p < 0.001). The main effect of exercise protocol was also significant after adjusting by the influence of VR and the interaction with VR (p < 0.001).

## Discussion

The VR-driven exercise games have increasingly used in rehabilitations in patients and elderly populations. Implementing games in lie-down posture may be beneficial to the adults who were bed bounded or with limited activity space. This study examined the influence of exercise protocols (in terms of set-interval and repetition-interval rest time) on the cardiopulmonary measures, blood lactic acid level, and participant’s experience during a recumbent-boxing exercise. Our experiment has worked on middle-aged adults, in an attempt to promote an active healthy lifestyle/habits at their pre-older and older ages later. Nevertheless, different age groups might have different preferences of gaming elements and display types that shall be considered.^49^ Besides, our research found that exercise protocols with different set-interval and repetition-interval rest times could significantly affect most of the cardiopulmonary measures as well as the overall blood lactic acid level. There was an apparent trend that drawing out the set-interval and repetition-interval rest time increased the average heart rate. The reduction of repetition-interval rest time alone could also level the MVV, oxygen consumption, and respiratory quotient. The increase of lactic acid indicates the degree of exercise intensity across various exercise protocols.^50^^,^^51^ Although VR attenuated the relationship between exercise protocol and the overall lactic acid level, our results suggested that VR did not impose further beneficial effects on the biomarkers. Thematic review also reported that VR exergame might provide level of exertion equivalent to that of real-world exercises.^52^

Subjective experience on VR demonstrated opposite findings to our original hypothesis, which suggested that VR is more interesting and engaging notwithstanding no significant enhancement on physical training effects. The participants in the VR group perceived lower interest, competence, and choice toward the interventions with increased level of pressure and effort. By virtue of the negative findings, we made follow-up phone calls to collect feedback informally from the VR group participants. Further analysis of responses by the VR group participants was carried out; half of them reported a negative experience toward the VR exergame, but another half of them reported that the VR exergame affected their physical training and performance. The major complaint was about the nuisance of the VR upon over-exertion of the physical training. With increasing exertion during the physical training, it was reported that the cheering crowd seemed to become noisier and sickening, while the screen-shaking effect increased with fatigue and cause dizziness. One previous study found that playing exergame while sitting may have caused a higher level of motion sickness than playing while standing.^44^ The interaction between exertion and posture on motion sickness is worth further investigation. Another complaint was about the game design. Controlling a uniform boxing speed on the opponents without other appealing features was very boring, especially during the strenuous exercise. In addition, the participants complained that the physical training was too intense and barely reachable. Teixeira et al.^53^ commented that over-exertion or out-performing previous performance shall be achievable to maintain intrinsic motivation.

Some other VR exergame-related research also reported the similar negative or ineffective findings.^54^^,^^55^ Some participants reported that VR games in sports are getting boring when the same scene or effects were repeated. VR games were reported to cause more dizziness with higher level of exertion, and the participants attempted to avoid eyesight to obviate the discomfort.^56^ On the other hand, some previous literature also found that the physical effect of VR was generally comparable to traditional interventions without the gear. Ersoy and Iyigun^57^ found that virtual and non-virtual boxing training produced similar training effects to post-stroke patients. The VR-simulated spaceflight exercises were no better than the controlled exercises but demonstrated better motivation, affection, and mood restoration.^58^ A meta-analysis suggested that extended reality (X-reality) interventions produced equal performance enhancement compared to non-virtual environment.^59^ Nevertheless, there were a number of positive reports with the integration of VR and high-intensity exercises. Williams et al.^60^ improved performance of long-distance cyclists by VR and believed that could be attributed by distraction deterring exertion. Farrow et al.^61^ implemented a HIIT on ergometry with a VR environment to race against own performance. The outcome was positive, and the participants developed motivation to “beat the person you were yesterday.” In addition to reduced training time and altered perception of intensity, resistance training methods that have varying rates of efficiency are known to promote divergent metabolic and endocrine responses.^50^ In the future, the use of VR technology could help to optimize training time, perception, and training protocol to give appropriate metabolic, neuromuscular, and endocrine responses.

To our best knowledge, this is the first study to examine the VR-induced exercise intervention to improve the aerobic capacity in the bed-bounded individuals. In clinical setting, some patients with lower limb surgery and disability may need to maintain their fitness and muscle strength during bed-bounded state for at least one month. Some older and weaker patients need to achieve the basic cardiorespiratory fitness before the surgery can be carried out.^62^^,^^63^^,^^64^ In microgravity application, the astronauts may strive for the moderate-to-high exercise intensity to improve the upper limb strength during the limited available time in spaceflight mission.^65^^,^^66^ Although the VR exercise group did not reveal the further training benefits over the non-VR exercise group, the VR boxing exercise in our study can achieve the moderate-to-high-intensity training effect in bed-bounded posture, which cannot be achieved by the existing hand cycling protocol.^67^^,^^68^ Future study should investigate the effects of various VR contents such as boxing, baseball, and karatedo on exercise prescriptions for bed-bounded populations.

### Limitations of the study

Some limitations have to be considered when interpreting our results. First, only the middle-aged healthy participants were recruited in this pilot study. The next step should adopt and optimize the VR boxing exercise protocol to the weaker and older participants who are actually bed bounded. Second, the VO_2_max was continuously monitored throughout each exercise period, which may affect the subjective scores on the exercise protocols. Thirdly, we have not collected information about the user preferences and acceptance toward VR and wearable device (e.g., controllers). These factors affect the perceived ease of use, usefulness, and, thus, intention to persist and the training effects, especially for older adults.^69^^,^^70^ Finally, the hit scores of the boxing exercises were quantified with the algorithm developed by the Exergame company. The optical motion capture approach should also be integrated into the system in order to quantify the movement quality across different boxing exercise protocols.

## STAR★Methods

### Key resources table

**Table:** 

REAGENT or RESOURCE	SOURCE	IDENTIFIER
**Deposited data**		

De-identified raw data of experiment	Github	https://github.com/BME-AI-Lab/virtualrealityboxing

**Software and algorithms**		

R program and studio	R Foundation	https://www.r-project.org/
Graphpad Prism 9	Dotmatics	https://www.graphpad.com/
Virtual Fighting Championship	L&L Technology PTY LTD	https://www.vfcgame.com/ and can be downloaded at https://store.steampowered.com/app/779130/Virtual_Fighting_Championship_VFC/

**Other**		

Virtual Reality Headset and Controller (PICO Neo 3 Pro)	PICO Immersive Pte. Ltd.	https://www.picoxr.com/global/products/neo3-pro-eye
Heart Rate Monitor (Polar 5 Pulse)	Polar	https://www.polar.com/en/products/heart-rate-sensors/
Metabolic Masurement Device (Metalyzer 3B)	Cortex Biophysik GmbH	https://cortex-medical.com/EN/METALYZER-3B-en.htm
Lactic Acid Analyzer (Biosen C-Line Gluocise and Lactate Analyzer)	EKF Diagnostics	https://www.ekfdiagnostics.com/biosen-analyzer.html

### Resource availability

#### Lead contact

Further information and requests for resource could be direct to the corresponding authors, Qiuhong HE (00002826@cczu.edu.cn) and Duo Wai-Chi Wong (duo.wong@polyu.edu.hk).

#### Materials availability

The study did not generate new unique reagents.

### Experimental model and subject details

#### Study design

This research was a balanced randomized controlled crossover study. Recruited patients were randomly assigned into the VR (with boxing-exercise content) and non-VR (with only white plain screen) groups. All participants in both VR and non-VR groups performed six sets of boxing-exercise protocols presented in a randomized order. Training effects were evaluated with cardiopulmonary measures and blood lactic acid levels. Questionnaire were also used to evaluate subjective experience.

#### Participant recruitment

Fifty-two healthy middle-aged male participants were recruited in this study with convenient sampling from local community. All participants reported they were physically fit and had exercised regularly. Inclusion criteria were: 1) age 40 – 50; 2) perform regularly moderate intensity physical exercise at least 5.5 hours per week:^71^ 3) perform physical exercise at least two times a week; 4) had at least 3 years of physical exercise experience. They shall have no previous trauma or surgery on the upper limb. Two of the participants quitted during the experiment period because of their pre-existing health problems (asthma and allergic rhinitis). Therefore, a total of 50 participants completed the experiment.

A baseline assessment was conducted to compare VO_2_max between groups using the Bruce protocol (multistage exercise treadmill test). In brief, participants ran on the treadmill for 21 minutes. They started with gentle and submaximal stress running but gradually progressed to increasing workload. For every three minutes, the running speed and treadmill inclination increased to induce exhaustion.^72^ VO_2_max and MVV were measured by a face mask and valve system (Cortex-Metalyzer-III, Cortex Biophysik GmbH, Leipzig, Germany). The total duration of the baseline assessment was about three hours.

The VO_2_max level was confirmed if three out of the four criteria were met:^73^ 1) > 180 heartbeats per minute; 2) respiratory quotient reached or approaching 1.15; 3) a plateau in oxygen consumption; 4) reached the maximum exertion level (i.e., RPE > 19). Moreover, the test was regarded as unsuccessful if the oxygen exchange rate was less than 1.1. If the baseline test failed, the participants repeated the baseline assessment on another day. In addition, the assessment would be terminated if the heartbeat, blood pressure, and ECG appeared abnormal or the participants reported or were observed sick during the test. The results from the baseline assessment confirmed no ineligible participant found and discarded from the study.

The study was approved by the institutional review board of the university (reference number: ZZUIRB2022013) and the Chinese Clinical Trial Registry (reference number: ChiCTR2300072656). All participants signed an informed consent after receiving oral and written descriptions of the experimental procedure before the experiment. Participants received incentives successfully completing all experimental tasks and received additional incentives for reaching the top 10 of both groups to encourage maximum exertion and performance during exercise interventions.

### Method details

#### Research protocol

After the baseline assessments, we randomly assigned 50 participants into VR and non-VR groups with an equal ratio (i.e., 25 per group). Then, participants in both groups performed six different exercise protocols in a randomized sequence, on separate days with about 48 hours apart. The randomization for group allocation and sequencing were generated using a computer program. Figure 4 illustrates the experimental protocol of the study.

The six exercise protocols involved the combination of two set-interval and three repetition-interval rest time. Set-interval rest time was either set at 0 second or 40 seconds, while repetition-interval rest time was set at either 0 second, one-third seconds, or two-third seconds (denoted as: S_0_R_0_, S_0_R_1/3_, S_0_R_2/3_, S_40_R_0_, S_40_R_1/3_, and S_40_R_2/3_). Both set-interval and repetition-interval rest time were the key controlling factors to adjust the training intensity and the degree of aerobic training. Shorter rest times induce stronger training effects and challenge the aerobic system. All exercise protocol conditions involved six sets of exercises with 120 punches (reps), which were 720 punches in total. The total time spent on each exercise protocol and post-exercise assessment was less than an hour. Table 2 details the design of the six exercise protocols included the average punch speed and the total duration of the task. Both VR and non-VR groups performed the identical exercise protocols, except for the VR content presented in the VR headset (boxing exercise content vs. green blank content).

#### Experimental setup and intervention

Figure 1 illustrates the experimental setup of the participants. The participants lay supine supported by a hanged frame with hardness over the head-torso region. A soft mattress was placed under the participants for sake of safety. The load resistence was estimated by the forearm weight times forearm length divided by two. The participants of the VR and non-VR groups put on a commercial available VR headset and controller (PICO Neo 3 Pro, Brand and Series of Headset, PICO Immersive Pte, United States) that provides improved accurate and latency experience, but the game scene was not presented (i.e., green plain screen) for the non-VR group. The VR headset featured a six degree-of-freedom wearable head-mounted display and two hand-held tracking controllers (PICO Neo 3 Pro, Brand and Series of Headset, PICO Immersive Pte, United States) with inside-out lighthouse tracking. The head-mounted display utilized a display at resolution of 1832 ×1920 per eye, a refresh rate of 90 Hz, and a field-of-angle of 90°. The headset also equipped with audio speakers and 3.5 mm audio jack.

The boxing exercise protocol and the game theme were designed and developed by an interdisciplinary team of VR innovation and technology company (L&L Technology PTY LTD, Melbourne, Victoria, Australia) included computer engineers, biomechanists, sport scientists, and coaches. For the virtual environment of the boxing game, it was taken place in a boxing ring inside a stadium. The boxing ring was surrounded by crowd cheering and applause with audio feedback to the players. The participants chose to control a character and fight against a humanoid robot avatar (Figure 1). During the game, the participants faced to the opponents in the first-person view and their hand-held controller served as virtual fists in the game. A dashboard was displayed on the environment with the number of points, number of punches, and time.

The game started when the player pushed the “start” button using the hand-held controllers. The objective of the game was to punch and knocked down the opponent on-target as much as possible and aligns with the prescribed cadence of the exercise protocol (Table 2). The game engine enabled physics simulation. Once the opponent was punched, it knocked back and resembled human movement (ragdoll movement) with sound effects. Participants scored one point with a successive punch on the respective opponents in each exercise protocol.

#### Experimental procedure

Before joining the experiment, all participants watched a demonstration video to understand the experimental procedures. The participants were asked not to perform strenuous physical exercises 48-hour before the experiment. They shall not take caffeine or alcohol drink 24-hour before the experiment and fast (except water) 3-hour before the experiment, which was confirmed verbally. Before the start of the experiment, the participants lied in a supine position for 20 minutes to familiarize to the environment until their breathing rate, heartbeat, and blood pressure were steady. Both groups were arranged in the same setup. Next, the participants performed a set of warm-up exercises to get ready (10 alternating punches at one punch per second).

Both VR and non-VR groups wore the VR headset during the experiment but a blank screen in soft green colour was shown for the non-VR group. Then, the participants started and performed six sets of 120 punches (repetitions) boxing exercises according to the assigned exercise protocol of that visit.

Heart rate, oxygen uptake, and ventilation volume were measured continuously throughout the entire exercises, with polar heart rate monitor (Polar 5 Pulse, Polar, Kempele, Finland) and metabolic measurement device (Coretx Metalyzer-III, Germany). The data were extracted by excluding the rest time intervals. The participants reported the Borg Rating of Perceived Exertion (RPE)^74^ in each repetition-time interval and completed the Post-experimental IMI^75^ and SVS^76^ after each exercise protocol. Earlobe blood samples were collected immediately after the last repetition as well as 3 minutes, 5 minutes, 10 minutes, 20 minutes, and 30 minutes after the last repetition to measure the level of lactic acid (Biosen C-Line Glucose and Lactate Analyzer, EKF Diagnostics, Cardiff, Wales, United Kingdom).

### Quantification and statistical analysis

At the baseline, we conducted the independent sample t-tests to investigate whether there were significant differences on age, body mass index, VO_2_max, MVV, and oxygen pulse between the VR and non-VR groups. Oxygen pulse is defined as the ratio of oxygen consumption to heart rate.

Since there were multiple independent factors, we adopted a preplanned strategy that aligned with our research objectives to simplify the statistical analyses. For the first objective that evaluated the influence of exercise protocol, we only analyzed the data from the non-VR group. We extracted the cardiopulmonary measures, including average heartrate, oxygen consumption, respiratory quotient, MVV, and EPOC. The EPOC is defined as measurably increased rate of oxygen intake following strenuous activity and it clearly changes across the increase of exercise intensity.^77^ For the first objective, one-way ANOVAs with repeated measures were used to determine whether they were significant differences between exercise protocols on the cardiopulmonary measures, while the oxygen pulse and respiratory quotient were analyzed by the Friedman tests because these two variables did not fulfil the requirements of parametric tests. Post-hoc pairwise comparisons with paired t-test (or Wilcoxon Signed Rank test for non-parametric tests) were conducted with Bonferroni adjustment, when the omnibus tests were significant. On the other hand, the overall effects on the lactic acid levels in all timepoints were evaluated using the repeated measures in multivariate semi-parametric factorial design (MultiRM).

For the second objective that investigated the influence of VR intervention, a GEE were used to find any significant interactions and main effects of VR intervention on the cardiopulmonary measures and participant subjective experience (via questionnaire). The participant’s subjective experience was evaluated using the IMI,^75^ which consisted of seven constructs: interest/enjoyment, perceived competence, effort/importance, pressure/tension, perceived choice, value/usefulness, and relatedness. Each construct consisted of seven questions and could be rated from one (not at all) to seven (very true). Moreover, the SVS measures the level of vitality using seven questions that could be rated from one (not at all) to seven (very true).^76^ The points of both instruments ranged from 7 to 49. Furthermore, a the overall lactic acid level would also be evaluated using the MutliRM by adding the group and interaction factors.

### Additional resources

The study was registered in the Chinese Clinical Trial Registry (reference number: ChiCTR2300072656).

## Data Availability

De-identified data have been deposited at Github and are publicly available as of the date of publication. Link is listed in the key resources table. The paper does not report original code.
